# Late Subconjunctival Emphysema in an Unrepaired Orbital Floor Fracture

**DOI:** 10.7759/cureus.24459

**Published:** 2022-04-25

**Authors:** Emanuel F Boyer, Oliver Filutowski, Charles Slonim

**Affiliations:** 1 Department of Ophthalmology, University of South Florida Morsani College of Medicine, Tampa, USA

**Keywords:** orbital fracture, orbital floor fracture, subcutaneous emphysema, orbital emphysema, subconjunctival emphysema

## Abstract

A 42-year-old gentleman with a history of a left orbital floor fracture four years prior presented to the emergency department following a motor vehicle collision. He was without subjective eye concerns, although a physical examination revealed a superior temporal subconjunctival mass with crepitus of the left eye. Visual acuity was 20/20 bilaterally, pupils were reactive without a relative afferent pupillary defect, and extraocular movement was fully intact. A computed tomography scan of the face revealed left-sided subconjunctival, subcutaneous, and orbital emphysema determined to be associated with a previous orbital floor fracture. With no other medical concerns requiring immediate treatment, the patient was offered outpatient repair of the old orbital floor fracture.

## Introduction

Subconjunctival emphysema is an uncommon phenomenon most often produced by existing orbital emphysema in which air is forced into the soft tissues surrounding the orbit, characterized by the presence of air between Tenon’s layer and conjunctiva. Often, subconjunctival emphysema is obscured by more pronounced subcutaneous periorbital emphysema [1]. Although subconjunctival emphysema is uncommon, it is often seen in close association with orbital emphysema, the presence of air posterior to the orbital septum. In medial orbital wall fractures, up to 75% of patients demonstrate orbital emphysema [2]. While orbital emphysema is most frequently caused by traumatic injuries with acute orbital floor fractures, this is not the rule; multiple creative mechanisms have been described, many related to pressurized air [3-6]. Most cases resolve spontaneously without complication within days to weeks of presentation; however, permanent visual impairment is possible if orbital compartment syndrome occurs and tension is placed on the optic nerve [7]. In this report, we offer a unique presentation of a patient with a previous orbital floor fracture and new subconjunctival emphysema following a motor vehicle collision with airbag deployment.

## Case presentation

**Figure 1:**
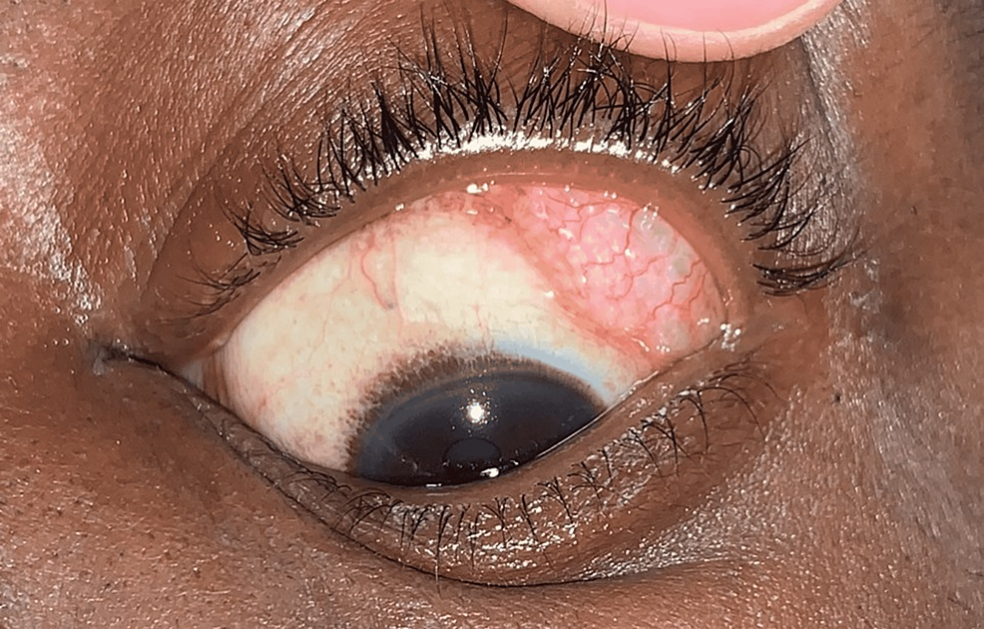
A freely mobile subconjunctival, cystic mass.

**Figure 2:**
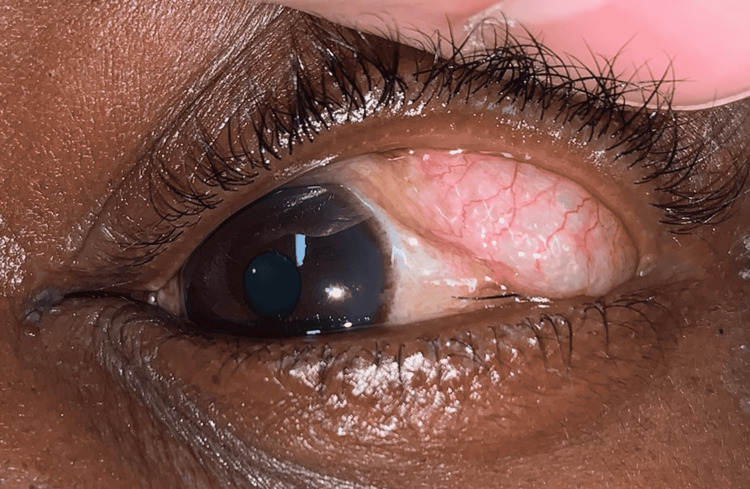
Appearance of subconjunctival emphysema with compression where crepitus could be felt.

A 42-year-old gentleman presented to the Emergency Department following a motor vehicle collision in which he was a restrained passenger and the front airbag deployed. His ocular history was significant for an unrepaired left orbital floor fracture sustained four years prior. On presentation, he reported no diplopia or visual changes. On gross examination, he was enophthalmic on the left relative to the right side with 2 mm of left upper eyelid ptosis. A small, superficial laceration was noted on his nasal bridge. Upon elevation of the left upper eyelid, a soft, freely mobile, subconjunctival, cystic mass was encountered in the superior temporal quadrant of his left eye (Figures 1, 2). Visual acuity was 20/20 OU, intraocular pressure was 10 OD and 11 OS, and ocular motility and retinal examination were normal.

**Figure 3:**
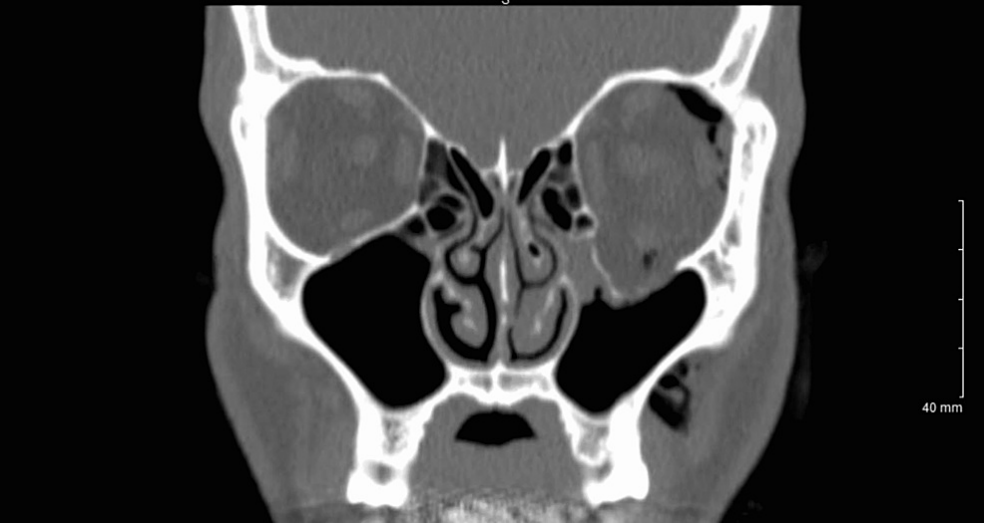
Blowout fracture of the left orbital floor with no blood products in the maxillary sinus. Orbital emphysema can be seen superiorly and inferiorly.

**Figure 4:**
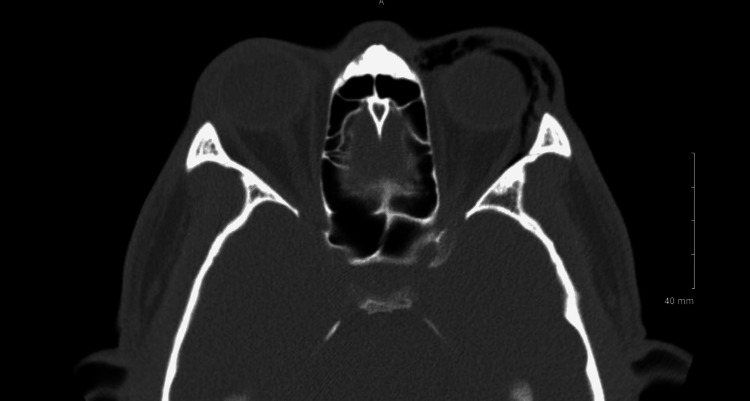
Subcutaneous, subconjunctival, and orbital emphysema can all be observed in this single axial section.

A computed tomography (CT) scan of the face revealed a large blowout fracture involving the majority of the left orbital floor without blood products in the maxillary sinus and with a focus of air within the orbit (Figure 3). Air could be seen tracking from the maxillary sinus into the orbit. Subcutaneous, subconjunctival, and orbital emphysema could all be observed in a single axial section (Figure 4). The absence of nasopharyngeal fractures on imaging attested that the soft tissue nasal bridge wound did not contribute to the orbital emphysema. There was no evidence of tension on the optic nerve.

After a thorough evaluation by the ophthalmology and ENT services, the structural findings related to the fracture were determined to be old due to an absence of blood products within the maxillary sinus. The patient elected for the outpatient repair of the orbital floor fracture. He was instructed to avoid blowing his nose until having his fracture repaired. The nasal bridge laceration was managed conservatively with topical antibiotic ointment. Unfortunately, the patient was lost to follow-up with our institution. Numerous unsuccessful attempts to contact the patient over the following six months were made.

## Discussion

In our review, there has been one prior report of a patient with simultaneous subconjunctival, subcutaneous, and orbital emphysema following an acute orbital floor fracture [8]. Prior studies have demonstrated that 20-60% of patients with an acute orbital fracture present with orbital emphysema [9,10]; however, the incidence of subconjunctival emphysema following orbital floor fracture has not been well established. In Ababneh’s report, the patient developed eye pain and proptosis following an episode of sneezing several hours after a fistfight. Several other reports of subconjunctival emphysema have been published in recent years, but these have generally been attributed to overtly pneumatic sources such as compressed air devices [11-13] or iatrogenic increases in intrathoracic pressure [3,14,15]. Our patient is unique in that he had no gross soft tissue bruising or swelling at the time of presentation; this suggests that his chronic unrepaired orbital floor fracture was the source of emphysema, rather than an acute injury seen in previously published reports. The abrupt increase in pressure from the motor vehicle collision and airbag deployment likely forced air from the maxillary sinus through communication in the unrepaired orbital floor facture into the orbit and surrounding tissues. Prior studies have suggested that in the absence of local trauma, subconjunctival emphysema can be explained by laxity between the subcutaneous and subconjunctival planes, allowing air to travel from the subcutaneous to the subconjunctival plane [1,16,17].

From the external examination, lymphangiectasias, chemosis, and prolapsed orbital fat may share certain characteristics with subconjunctival emphysema; the focal overlying vasculature of the subconjunctival air can be mischaracterized as a conjunctival soft tissue mass or an inflamed lacrimal gland. Orbital fat prolapse is seen radiographically as a continuous extension from the intraconal fat and would not extend across the anterior surface of the conjunctiva [18]. The hypolucent CT features, mobility on examination, and presentation after trauma all support the diagnosis of subconjunctival emphysema.

Management of subconjunctival emphysema is conservative as the air is typically absorbed over days to weeks without complication. Should the conjunctival swelling be so severe as to cause lagophthalmos, topical lubricants and taping of eyelids are typically sufficient; however, incisional conjunctival decompression can also be considered [3]. Parapharyngeal and retropharyngeal subcutaneous emphysema should be considered in patients with difficulty swallowing or labored breathing [19]. Permanent visual impairment is possible if associated with severe orbital emphysema. Canthotomy and cantholysis with possible needle decompression may be needed for patients with significant intraorbital air causing vision-threatening orbital compartment syndrome [7].

## Conclusions

While current literature describes subconjunctival emphysema following acute orbital trauma, to our knowledge, this is the first documentation of subconjunctival emphysema in a patient with a longstanding, unrepaired orbital floor fracture. This patient’s history predisposed him to having relatively unimpeded movement of air from his maxillary sinus to enter the orbital cavity and surrounding tissues. Given that this patient did not follow up for repair for the second time, recurrent subconjunctival, subcutaneous, and orbital emphysema is a real possibility should he sustain additional trauma.
